# Bringing function to the forefront of cell therapy: how do we demonstrate potency?

**DOI:** 10.3389/fimmu.2023.1226841

**Published:** 2023-07-11

**Authors:** Mark W. Lowdell, Ben Weil

**Affiliations:** ^1^ INmuneBio Inc, Boca Ratan, FL, United States; ^2^ Cancer Institute, University College London, London, United Kingdom; ^3^ Centre for Cell, Gene & Tissue Therapeutics, Royal Free London NHS Foundation Trust, London, United Kingdom

**Keywords:** ATMP, functional assay, quality control, commercialisation, bla, MAA

## Abstract

Unlike conventional pharmaceuticals, biologics and Advanced Therapy Medicinal Products (ATMPs) are required to meet a standard of “potency” as part of the final release criteria at completion of manufacture. During early phase clinical trials, most regulatory agencies have been willing to accept very immature potency assays with an expectation that these will be improved, qualified and validated during the clinical development of the drug to Marketing Authorisation Application (MAA) or Biologics License Application (BLA) submission.This model of continuous development of potency assay in parallel with drug development has already led to at least two notable problem cases; namely Iovance and Mesoblast. Both companies completed successful phase III clinical trials but, in both cases, the initial BLA was rejected on the basis that their potency assay for drug product release was inadequate. Fortunately these issues appear to have been overcome in March of this year, with Mesoblast receiving acceptance of their BLA for Remestemcel and Iovance obtaining a rolling BLA approval for Lifileucel.

## Introduction

Unlike conventional pharmaceuticals, biologics and Advanced Therapy Medicinal Products (ATMPs) are required to meet a standard of “potency” as part of the final release criteria at completion of manufacture. During early phase clinical trials, most regulatory agencies have been willing to accept very immature potency assays with an expectation that these will be improved, qualified and validated during the clinical development of the drug to Marketing Authorisation Application (MAA) or Biologics License Application (BLA) submission.

This model of continuous development of potency assay in parallel with drug development has already led to at least two notable problem cases; namely Iovance and Mesoblast. Both companies completed successful phase III clinical trials but, in both cases, the initial BLA was rejected on the basis that their potency assay for drug product release was inadequate. Fortunately these issues appear to have been overcome in March of this year, with Mesoblast receiving acceptance of their BLA for Remestemcel and Iovance obtaining a rolling BLA approval for Lifileucel.

Each company had been asked to present a potency assay for their drug which reflected the claimed mechanism of action. This seemingly innocuous demand delayed resubmission of both BLAs for many months and raised questions about how to demonstrate Mechanism of Action (MoA) for an autologous tumor-infiltrating lymphocyte product or an allogeneic mesenchymal stromal cell.

The rapid development of ATMPs has outpaced regulatory advice; the ICHQ6b standards for potency assays for biologics has not been updated since 1999 and provides little direction with regard to cell and gene medicines. The most recent Food & Drug Administration (1) document pertaining to potency assays for cell and gene medicines was published in January 2011 and was declared “current” in May 2019.

The challenges of testing ATMPs are well recognised and regulatory agencies around the world have started to consider standards for QC assays for ATMPs. In this article I will discuss the nature of “Potency”, but this assumption is predicated on the definition of potency of an ATMP; here regulatory advisory documents are very vague and somewhat contradictory.

## Definitions of “potency”

The FDA defines potency of biologics in 21 CFR 600.3(s) as “the specific ability or capacity of the product, as indicated by appropriate laboratory tests or by adequately controlled clinical data obtained through the administration of the product in the manner intended, to effect a given result.” In Europe, European Medicines Agency (EMA) defines potency as “the quantitative measure of biological activity based on the attribute of the product, which is linked to the relevant biological properties”. Helpfully, EMA goes on to say, “The assay demonstrating the biological activity should be based on the intended biological effect which should ideally be related to the clinical response. Preferably, the potency assay should reflect the clinical Mechanism of Action”.

From the statements above, perhaps an appropriate harmonised definition is “potency is a confirmation that the batch of drug can perform the mechanism of action claimed for the drug”.

However, ATMPs are complex products which are difficult to characterise; determining the mechanism or mechanisms of action may not be straightforward. Even a single claimed MoA may require a combination of multiple analytes and, when developing a product with more than one MoA, multiple methods will be needed to assess potency. In the common example of a CD19-CAR-T cell product, the CD19-specific lysis of a model lymphoma cell line is an easily defendable claimed MoA. The chosen assay and acceptance criteria might be more difficult to defend but we will discuss those later. Nonetheless, from a scientific perspective, CAR-T cells not only lyse target cells directly, they also proliferate, engraft and secrete inflammatory cytokines which activate other CAR-T cells in the infusion as well as the endogenous T, B and NK cells, resident dendritic cells, neutrophils and macrophages leading to multiple secondary and tertiary effects, some of which may be adverse.

These multiple potential MoA will arise during drug development and it should be recognised that different assays may be needed for different stages of development and of manufacture. Not all potency assays may be suitable for product release, but they may be valuable in showing product consistency during process changes or stability testing. It is important to understand the setting in which a specific assay will be used and whether the regulatory expectations will be different; generally, in-process QC assays and measures of comparability during manufacturing process development are less demanding than release assays used for batch approval.

Having decided upon MoA the next stage is to determine the appropriate analyte(s) to measure. To use the example above, a CD19 CAR-T may function through:

1. direct lysis of CD19+ tumour cells through secretion of granzymes and perforins
2. indirect lysis of CD19+ tumour cells through TRAIL or FAS mediated induction of apoptosis
3. secondary immune activation through release of proinflammatory cytokines.

The first two can be measured by cytotoxicity assays using a CD19+ cell line such as RAJI, but the choice of assay and controls of that assay must be considered and have been reviewed recently (2). Functional assays have large coefficients of variance (cv), often >30% making, them challenging as product release assays at the commercialisation stage. Also, they are time consuming and difficult to semi-automate, which makes them difficult to apply to a commercial operation releasing thousands of autologous batches per year. Perhaps a cell cytotoxicity assay is the best during drug development and for control of manufacturing changes, but may be not for drug product release? If it remains the chosen release assay, how can it be adequately controlled?

We are developing a commercial ATMP which requires a functional tumour lysis assay for final batch release. This is a Natural Killer (NK) cell priming product and a relevant target cell is RAJI. We have recognised that RAJI cells have differential susceptibility to NK lysis dependent upon their duration in continuous culture and have decided to create a master cell bank of RAJI which we have accredited as “true” RAJI cells. We have produced a working cell bank and aliquots of seed vials for each potency assay we run. Each assay requires thawing of a vial and initiation of suspension culture 24 hours prior to the assay to ensure that the RAJI cells are in exponential growth phase at the time of the assay. Using this approach and a flow cytometric cytotoxicity assay perfected over several years, we can demonstrate inter-assay cv of <10% and intra-assay cv of <20%. However, the critical issue of relevant control reagents remains a challenge. In the CD19+ CAR-T example above, one option for a positive control could be a CD19+ CAR-T from a single healthy donor apheresate which is manufactured, aliquoted and stored. A vial can be thawed and used with every QC test run. When the donor batch is close to running out, a new donor apheresate can be acquired and used to make a new QC reagent after parallel testing against the existing standard, creating a new acceptance threshold for batches of drug. This approach is used by at least one commercial CAR-T developer, but the stability of the control CAR-T cells over their lifespan must be determined during drug development, which is another challenge to meet and cost to be managed.

Functional target cell lysis assays are very attractive during drug development and early clinical trials, but the duration of the killing assay must be optimised for the chosen target cell and specific assay platformed. There are multiple ways to measure target cell lysis directly and indirectly depending on the chosen target cells; all require a way to use the data to determine a single value to act as the acceptance criterion for assay performance (positive control) and for the drug product as suitable for release. Most cytotoxicity assays use a range of effector:target cell ratios and resolving those into a single result can be difficult. Devices such as the xCelligence platform (Agilent Technologies) and the IncuCyte (Sartorius) are valuable when using adherent tumour cells as targets and can measure cytotoxicity at a variety of E:T ratios and over prolonged culture periods. The resulting complex data can be reduced to the total killing over time for each E:T ratio tested by calculating the area under the curve (AUC) and then calculating the mean AUC across the different E:T ratios. This can provide a very robust assessment of lytic potency of a cell product.

If not using a target cell lysis assay, it may be possible to use flow cytometric measurement of surface TRAIL or FAS, or intracellular expression of granzymes/perforins/cytokines after ligation of CD19 CAR with a recombinant CD19 multimer reagent. This could avoid the need for banks of RAJI cells but a healthy donor CAR-T control product would still be a valuable “positive” control for the assay.

Cytokine synthesis or secretion are common potency assays for ATMPs, measured by flow cytometry, ELISA or bead arrays. Assay CVs are typically low and it is often relatively easy to propose a MOA based upon cytokine release. However, given the breadth of possible MOA for a typical CAR-T product, it is likely that no single assay will describe total potential “potency” but maybe one assay can be used as a surrogate?

Therapeutic anti-CD20 monoclonal antibodies such as Rituximab are biologics and require potency testing as part of their release criteria. The claimed MOA of Rituximab were CD20-mediated complement-dependent lysis (CDC), antibody-dependent cellular cytotoxicity (ADCC) and antibody-dependent cellular phagocytosis (ADCP). The potency assay approved by (3) Center for Drug Evaluation and Research (application number 761103Orig1s000) was a single assay for CDC using a CD20+ tumour cell line (4); the other mechanisms of action were not tested.

## Does “potency” require a functional assay?

A common perception among ATMP developers is that a potency assay must involve a measurement of cell function *in vitro*, which often leads to development of assays of cell functions which bear little relationship to the MOA of the product. *In vitro* lysis of CD19+ Raji cells in the CAR-T example above is a good example of a functional potency assay which has direct relationship with the proposed MOA. However, during development of the CD19-CAR-T, it is likely that data were acquired on the number of CD19-CAR molecules required to trigger the T cell. An alternative potency assay could, perhaps, be a combination of the median number of CAR molecules per cell and the viability.

The conventional tumour cell killing assay requires the establishment and maintenance of a relevant target cell line; ideally with a master cell bank, working cell banks, continuous testing for identity and sterility, and minimum/maximum number of passages at the time of use. This represents a considerable burden even before validation of the equipment and the assay.

In some settings a functional assay which reflects the drug MOA is technically impossible. Preparations of autologous dendritic cells (DC) have been used in multiple clinical trials, mostly to stimulate an acquired immune response to tumours but, in a small number of cases to induce tolerance in an autoimmune disease setting.

## Establishing “acceptance criteria”

Having chosen and qualified a potency assay or portfolio of assays, the most difficult step is setting the acceptance criteria for each assay. Within our experience, we have released autologous CD19 CAR-T cell products on the basis of *in vitro* lysis of CD19+ RAJI cells, but the question remains, “how much lysis is *potent*?” or “how many cytokine secreting cells are needed?”.

The complexity of ATMP function *in vivo* is well recognised but incompletely understood, especially in the setting of cellular immunotherapy of solid tumours or immunosuppressive effects of mesenchymal stromal cells. As the ATMP approaches the end of drug development through clinical trials, the temptation arises to go back to clinical trials data and see if there were batches associated with successful treatment and others that failed to show clinical benefit, and look for potency assay data which could have identified the “potent” versus the “non-potent” batches; i.e. “if my batch of drug meets this potency criterion then it will have a clinical effect *in vivo”*. This is a uniquely high barrier for any drug at completion of manufacture and conflates data from GMP with GCP data acquired during the clinical trials.

Indeed, although some groups have shown correlations between *in vitro* lytic function of CAR-T products with *in vivo* efficacy in animal models (5), studies of CAR-T cell trials have shown that cytokine release and *in vitro* tumour lysis assays do not predict clinical efficacy (6), even for CD19-CAR-T targeting haematologic disease. Adoptive cell therapies targeting solid tumours, whether they are tumour-infiltrating lymphocytes, CAR-T, CAR-NK, anti-tumour macrophages or neutrophils all require the ability to migrate into the tumour site and this will be a critical functional criterion, yet one which is uniquely difficult to test. Thus the *in vivo “*failure” of some batches is likely to be multifaceted; was success versus failure due to:

1. degree of engraftment of the ATMP which is as much a measure of successful lymphodepletion as drug potency, and certainly not measured by a target cell killing or cytokine secretion assay
2. the dynamics of ATMP cell expansion versus the replication of the specific tumour or the inflammatory immune response being treated
3. the shedding of target antigens by the tumour
4. and many more

Using clinical outcome from trial subjects may not be an appropriate criterion to set an acceptance level of potency. In the scenario of the CD19-CAR-T, the claimed MOA of the CAR-T is that it kills CD19+ lymphoma cells. A suitable acceptance criterion could be that the CAR-T product lyses more CD19+ cells that the non-transduced T cells from the same donor. Here the definition of “more than” could be based upon a statistical test of significance using triplicate (or more) replicates. Once a suitable acceptance criterion has been set, one can then identify the appropriate assay and seek to validate it to the standards of ICH Q2(R2).

It is easy to get buried in the immunobiology of our products and conflate concepts of *in vivo* functional scenarios with the definition of MOA. In such a setting, perhaps our potency assay for an autologous CD19 CAR-T should combine an assay of lysis of a CD19+ tumour cell with release of T cell-derived cytokines. Should we measure IFN-g and TNF-a secretion alone or should we include IL-1, IL-10, IL-13 IL-17, IL-22 and IL-26 as known T cell derived cytokines which we can postulate have roles in CAR-T efficacy? Plainly there is no simple answer to this question and it is unlikely that a regulatory agency reviewing a trial application or a marketing approval would be in a position to determine which of these analytes is appropriate for an individual product. The responsibility lies with the drug developer to determine analytes which are scientifically justified and which can be measured reliably, reproducibly and accurately such that each assay can meet the standards of ICHQ2 with respect to:

- Linearity
- Accuracy
- Precision
- Reproducibility
- Specificity
- Range

As presented by Dr Shree Joshi to the US Pharmacopoeia (7) (https://www.usp.org/sites/default/files/usp/document/events-training/04-approaches-to-potency-testing-for-chimeric-antigen-receptor-t-cells-shree-joshi.pdf), an ideal potency assay should be:

- QC friendly
- A reflection of MOA
- Validated to ICHQ2
- Transferable to a CDMO or multiple manufacturing sites

## Conclusions

Scientifically it is valuable to gather as much data as possible and this is critical during drug development from pre-clinical concept through to the end of the registration trial, to provide data for commercial registration. The interpretation of these data should generate a full understanding of the MOA of the drug, from which potency assays can be conceived and tested through the clinical trial stages. It is essential to maintain a focus on which assays are suitable for different stages of manufacture and which could be used for commercial product release. From a regulatory perspective it is important to gather data which can be easily interpreted to provide a definitive answer with respect to drug quality, both as release criteria but also for stability testing of the drug product, to support the shelf-life assigned to the final drug product in the final drug packaging and in real-world storage conditions.

ATMPs are at the forefront of developmental medicines, precisely where monoclonal antibodies and recombinant cytokines were 30 years ago. ATMPs are far more complex than other biologics yet their speed of development has been faster and continues to be so. The questions of quality control and product definition persist, and regulatory guidance throughout drug development remain essential, but the drug developer can facilitate this by recognising that drug characterisation and drug definition, including potency, are different; the latter deriving from the former. It is important to plan for success and drug developers should characterise the drug during pre-clinical development and clinical trials to create a defendable definition at the time of submission for marketing approval.

## Data availability statement

The original contributions presented in the study are included in the article/upplementary material. Further inquiries can be directed to the corresponding author.

## Author contributions

Provided data, wrote part of the manuscript and proof read/corrected the final draft. All authors contributed to the article and approved the submitted version.

## References

[1] FDA. Guidance for industry – potency tests for cellular and gene therapy products. Available at: http://www.fda.gov/BiologicsBloodVaccines/GuidanceComplianceRegulatoryInformation/Guidances/default.htm.

[2] Kiesgen S Messinger JC Chintala NK Tano Z Adusumilli PS. Comparative analyses of assays to measure CAR T cell-mediated cytotoxicity. Nat Proc (2021) 16:1331–42. doi: 10.1038/s41596-020-00467-0 PMC8064272 33589826

[3] FDA. Center for drug evaluation and research (application number 761103Orig1s000). Available at: https://www.accessdata.fda.gov/drugsatfda_docs/nda/2019/761103Orig1s000ChemR.pdf.

[4] Chand S Kumar B Prathap VM Singh S Mahajan RV. Quality assurance of rituximab (anti-CD20) antibodies by potency testing: determining the system suitability criteria and sample acceptance criteria. Curr Sci (2018) 114:2513–8. doi: 10.18520/cs/v114/i12/2513-2518

[5] Qintarelli C Locatelli F Caruana I De Angelis B. Overcoming challenges in CAR T-cell product cGMP release. Mol Ther (2016) 24:845–6. doi: 10.1038/mt.2016.72 PMC4881777 27198849

[6] Li Y Hou Y Yu L Wang J. Quality control and nonclinical research on CAR-T products: general principles and key issues. Engineering (2019) 5:122–31. doi: 10.1016/j.eng.2018.12.003

[7] Joshi S. Approaches to potency testing for chimeric antigen receptor T cells. US pharmacopoeia biologics stakeholder forum (2022). Available at: https://www.usp.org/sites/default/files/usp/document/events-training/04-approaches-to-potency-testing-for-chimeric-antigen-receptor-t-cells-shree-joshi.ptpdeldf.

